# Neuroprotective effects of Canagliflozin: Lessons from aged genetically diverse UM‐HET3 mice

**DOI:** 10.1111/acel.13653

**Published:** 2022-06-15

**Authors:** Hashan S. M. Jayarathne, Lucas K. Debarba, Jacob J. Jaboro, Brett C. Ginsburg, Richard A. Miller, Marianna Sadagurski

**Affiliations:** ^1^ Department of Biological Sciences, IBio (Integrative Biosciences Center) Wayne State University Detroit Michigan USA; ^2^ Department of Psychiatry and Behavioral Sciences University of Texas Health Science Center San Antonio Texas USA; ^3^ Department of Pathology and Geriatrics Center University of Michigan Ann Arbor Michigan USA

**Keywords:** canagliflozin, hippocampus, hypothalamus, insulin, longevity, metabolism

## Abstract

The aging brain is characterized by progressive increases in neuroinflammation and central insulin resistance, which contribute to neurodegenerative diseases and cognitive impairment. Recently, the Interventions Testing Program demonstrated that the anti‐diabetes drug, Canagliflozin (Cana), a sodium‐glucose transporter 2 inhibitor, led to lower fasting glucose and improved glucose tolerance in both sexes, but extended median lifespan by 14% in male mice only. Here, we show that Cana treatment significantly improved central insulin sensitivity in the hypothalamus and the hippocampus of 30‐month‐old male mice. Aged males produce more robust neuroimmune responses than aged females. Remarkably, Cana‐treated male and female mice showed significant reductions in age‐associated hypothalamic gliosis with a decrease in inflammatory cytokine production by microglia. However, in the hippocampus, Cana reduced microgliosis and astrogliosis in males, but not in female mice. The decrease in microgliosis was partially correlated with reduced phosphorylation of S6 kinase in microglia of Cana‐treated aged male, but not female mice. Thus, Cana treatment improved insulin responsiveness in aged male mice. Furthermore, Cana treatment improved exploratory and locomotor activity of 30‐month‐old male but not female mice. Taken together, we demonstrate the sex‐specific neuroprotective effects of Cana treatment, suggesting its application for the potential treatment of neurodegenerative diseases.

## Graphical abstract

Our findings demonstrate sex‐specific neuroprotective effects of Canagliflozin treatment that are associated with reduced neuroinflammation, improved central insulin sensitivity, and locomotor activity in aged mice.

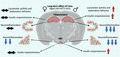

**Abbreviations**
17aE2: 17‐a‐estradiol
ACA: acarbose
ARC: arcuate nucleus of the hypothalamus
Cana: canagliflozin
CNS: central nervous system
mTOR: mechanistic target of rapamycin
SGLT2i: sodium‐glucose transporter 2 inhibitor

## 1 INTRODUCTION

The NIA‐sponsored Interventions Testing Program (ITP) was designed to identify therapeutic interventions that slow aging in mice, as assessed by increased lifespan, and has identified several treatments that successfully extend lifespan in the genetically diverse UM‐HET3 mouse model (Miller et al., 2007; Nadon et al., 2008; Harrison et al., 2014; Harrison et al., 2019). The ITP recently reported that an FDA‐approved anti‐diabetes drug, Canagliflozin (Cana), a sodium‐glucose transporter 2 inhibitor (SGLT2i), extended the median survival of male mice by 14%, without an effect on lifespan in females (Miller et al., 2020). UM‐HET3 mice are not prone to diabetes, and most deaths are attributable to various forms of neoplasia, suggesting that tumorigenesis was inhibited or decelerated by this drug, though only in males. Interestingly, Cana led to lower fasting glucose and improved glucose tolerance in both males and females, and diminished fat mass in females only (Miller et al., 2020). Sex‐specific lifespan benefit was also seen in male mice given another anti‐diabetes drug, Acarbose, which also reduces blood glucose by slowing the breakdown of carbohydrates in the intestine (Lam et al., 1998; Harrison et al., 2014). However, it is yet to be determined whether reductions in blood glucose per se are responsible for lifespan extension.

Improving glucose regulation and metabolic control through pharmacological interventions has been shown to be beneficial in delaying some aspects of aging (Gonzalez‐Freire et al., 2020). The cellular or molecular mechanism primarily responsible for this effect is not fully understood, although studies indicate that the interplay between lifespan extension and metabolic changes involves the ability to reduce systemic and/or central inflammation (Furman et al., 2019). For example, treatment of male mice with Acarbose or with 17‐α‐estradiol (17αE2), an optical isomer of 17‐β‐estradiol (Zhurova et al., 2009) extends lifespan preferentially in male mice only (Strong et al., 2016), reduces age‐associated metabolic and inflammatory dysfunction in the periphery and the brain (Garratt et al., 2017; Sadagurski et al., 2017). Similarly, the anti‐diabetes drug Metformin exerts its neuroprotective effects in aging by reducing neuroinflammation (Bharath et al., 2020; Kodali et al., 2021), although metformin appears not to increase lifespan in male or female mice tested by the ITP (Strong et al., 2016). Age‐associated neuroinflammation triggered by activated microglia and astrocytes induces neuronal stress that affects brain insulin signaling, cognitive impairment, and progression of neurodegenerative diseases (Bomfim et al., 2012; von Bernhardi et al., 2015).

Limited studies link SGLT2i treatment with neuroprotection. For example, the SGLT2i Empagliflozin reportedly reduced beta‐amyloid levels and improved cognitive abilities in a murine model of Alzheimer's disease (AD) crossed to the diabetes model of leptin receptor deficiency (db/db), or in db/db mice alone (Hierro‐Bujalance et al., 2020), (Lin et al., 2014). A recent clinical study demonstrated improved insulin sensitivity in the hypothalamus in subjects with prediabetes after treatment with Empagliflozin (Kullmann et al., 2021). Likewise, Cana reduced obesity‐associated neuroinflammation in the hypothalamus and nodose ganglion (Naznin et al., 2017). However, the underlying mechanisms of the beneficial effect of the SGLT2i were associated primarily with diabetes and obesity. The question of whether Cana has more general beneficial effects on the aging brain or protective effects from age‐associated neurodegeneration, in diabetes‐free mice, remains open. In this study, we evaluated the possible neuroprotective effect of long‐term Cana treatment in aged UM‐HET3 mice, with a specific focus on brain regions that are sensitive to metabolism and cognitive function.

## 2 RESULTS

### 2.1 Cana treatment improves central insulin sensitivity in aged mice

**FIGURE 1:**
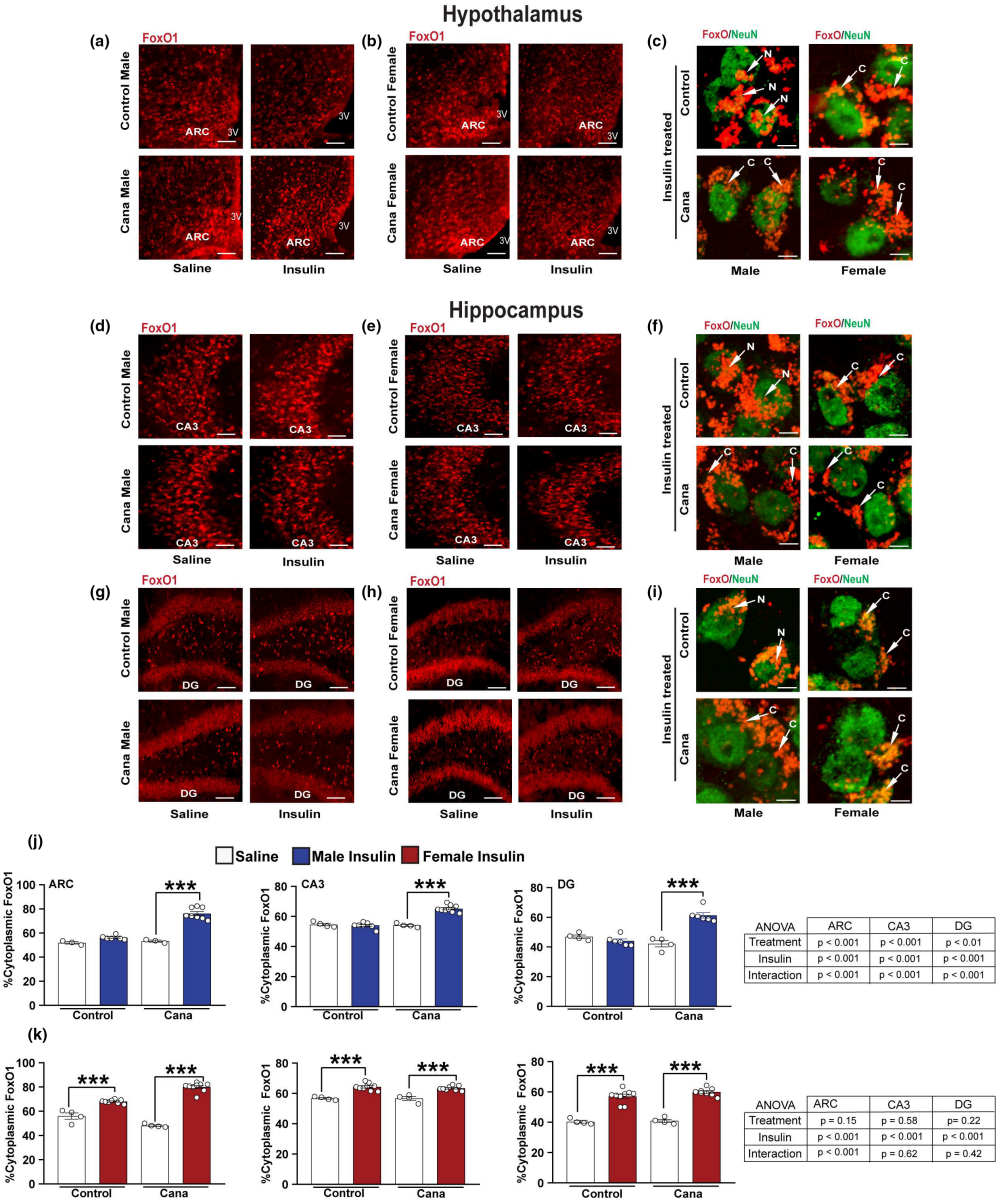
Insulin→FoxO1 signaling in aged Cana treated mice. Immunofluorescence for FoxO1 (red) and NeuN (green) in 30‐months‐old control and Cana treated mice injected with insulin (3 U/kg i.p.; 15 min) or saline. Representative images from the hypothalamus in males (a) and females (b), or hippocampus CA3 in males (d) and females (e), and dentate gyrus (DG) in males (g) and females (h) of control and Cana‐treated mice are shown. Scale bars represent 200 or 10 μm for the left panels for confocal images of insulin‐treated cells, showing FoxO1 (red) merged with NeuN (green) (right) in 30‐months‐old mice of the indicated groups (c, f, and i). White arrows indicate the localization of the FoxO1, C. cytoplasmatic, and N. nuclear. 3 V, third ventricle. Quantification of neurons containing cytoplasmic FoxO1 immunoreactivity in the ARC, CA3, and DG regions in males (j) and females (k); error bars show SEM for *n* = 4–7 mice/group, 3–4 images from each region/mouse were taken. Data were analyzed by 2‐factor ANOVA and further analyzed with the Newman–Keuls post hoc test (***p < 0.001). The tables demonstrate the two‐factor ANOVA analysis. Confocal images of higher magnification are shown in Figure S1

We have previously demonstrated that UM‐HET3 male and female mice treated with Cana from 7 months of age had significantly lower fasting blood glucose levels and showed an improved glucose tolerance by 22 months of age (Miller et al., 2020). To investigate whether Cana treatment affected central insulin sensitivity, we administered insulin intraperitoneally and assessed the distribution of the FoxO1 transcription factor between the nuclear and the cytoplasmic compartments of neurons in the hypothalamus and the hippocampus of 30‐months‐old control and Cana‐treated mice after 23‐months of Cana treatment. We found that long‐term Cana treatment sensitized the hypothalamic response to insulin, as shown by increased levels of cytoplasmic FoxO1 in the arcuate nucleus (ARC) of the hypothalamus in response to insulin administration in male mice. In contrast, old control males exhibited insulin resistance (sex x insulin interaction *p* < 0.01) (Figures 1a,c,j and Figure S1). A similar pattern in response to insulin stimulation was observed in the CA3 and DG sub‐regions of the hippocampus, with a significant interaction effect between Cana and insulin, only in males (sex x insulin interaction *p* < 0.0001) (Figures 1d,f,g,i and j). Both control and Cana‐treated females responded similarly to insulin stimulation with elevated cytoplasmic levels of FoxO1 in the hypothalamus and hippocampus without significant effect of Cana (Figures 1b,c,e,f,h,i, and k). To provide further evidence for Cana‐mediated changes in insulin signaling in the aging brain, we assessed the levels of phospho Akt (pAkt), a downstream mediator of the insulin receptor, upon insulin stimulation. The levels of pAkt increased significantly upon insulin stimulation in Cana‐treated males, but not control males, in the hypothalamus and the hippocampus, consistent with the FoxO1 cytoplasmic localization under these conditions (sex x insulin interaction *p* < 0.05) (Figures S2a,b,e,f,i, and j). As expected, pAkt levels were similarly upregulated in control and Cana‐treated female mice (Figures S2c,d,g,h,k and l). We did not detect differences in the expression levels of insulin signaling molecules including, *Irs1*, *Irs2*, and *Akt* in the microdissected hypothalamus from aged control and Cana‐treated animals (Figure S2m). These results are in accordance with previous reports indicating sex‐specific sensitivity to insulin action in the brain on food intake and body weight in human and rodent studies (Clegg et al., 2003; Hallschmid et al., 2004; Tramunt et al., 2020), demonstrating the protective properties of Cana treatment in the brain.

### 2.2 Cana reduces mTOR signaling in a sex‐specific manner in the hippocampus

**FIGURE 2:**
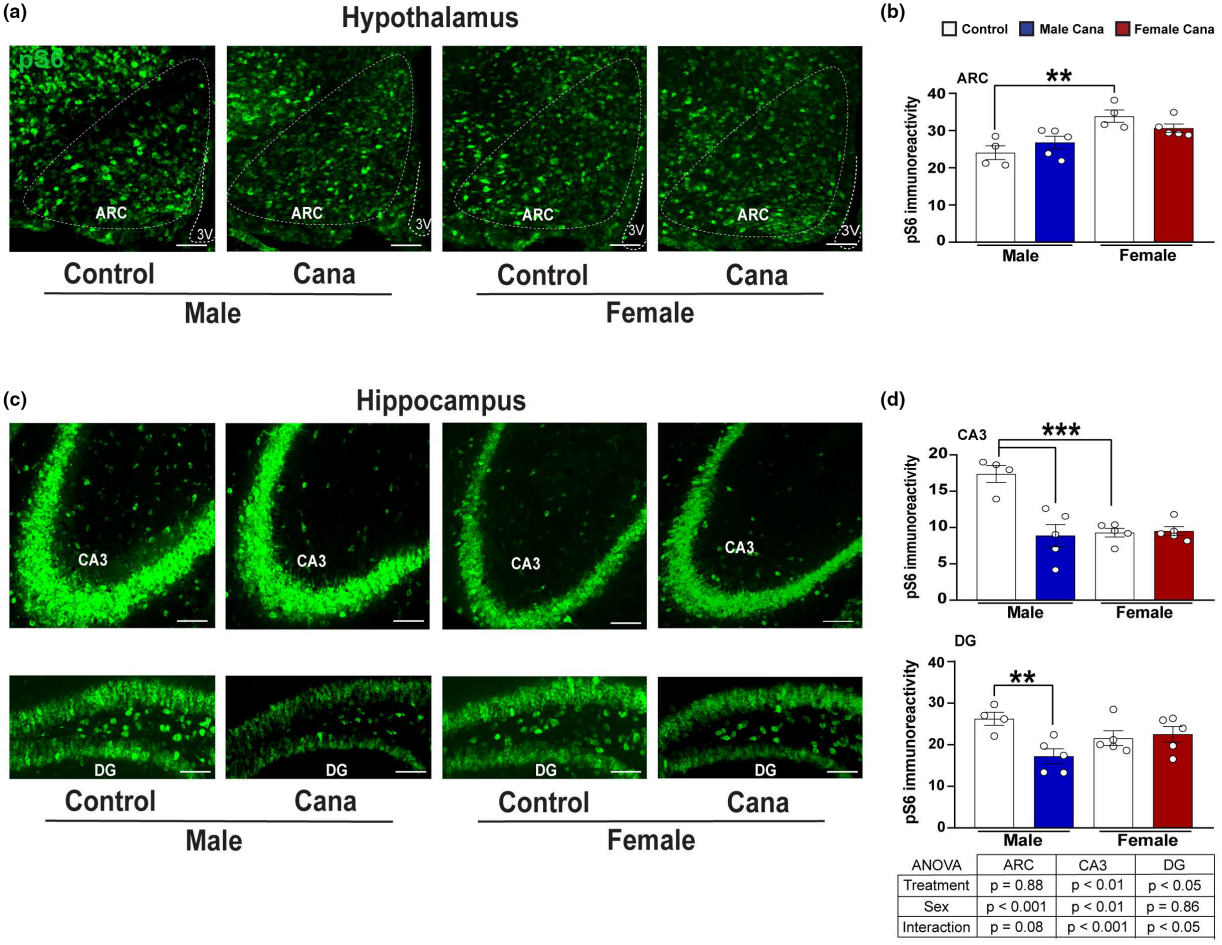
pS6 protein expression in aged Cana treated mice. Brain sections of 30‐months‐old male and female mice were analyzed for hypothalamic and hippocampal phosphorylated S6 protein expression. Representative images showing immunostaining in the arcuate nucleus of the hypothalamus (ARC) (a), hippocampal CA3, and dentate gyrus (DG) (c) of control and Cana‐treated male and female mice. Quantification of pS6 immunoreactivity in the ARC (b), CA3, and DG (d) of control and Cana‐treated male and female mice. Scale bars: 200 μm, 3 V, third ventricle; error bars show SEM for *n* = 4–5 mice/group. Data were analyzed by 2‐factor ANOVA and further analyzed with the Newman–Keuls post hoc test (**p < 0.01, ***p < 0.001). The tables demonstrate the two‐factor ANOVA analysis

Brain insulin resistance is associated with hyperactivation of the mechanistic target of rapamycin (mTOR). However, inhibition of the mTOR complex 1 (mTORC1) increases animals' lifespan (Lamming et al., 2012; Papadopoli et al., 2019). We assessed the phosphorylation state of S6 kinase (pS6, S240/S244), a downstream substrate of mTOR, in the hypothalamus of 30‐month‐old mice treated with Cana. We found a significant effect of sex, with females expressing higher levels of pS6 than males (*p* < 0.001), but there was no effect of Cana treatment on pS6 protein levels (Figure 2a, b). We detected significant sex‐specific effects in mice fed the control diet, where females expressed higher levels of pS6 in the hypothalamus (*p* < 0.01), while males expressed higher pS6 in the hippocampal CA3 sub‐region as compared to females (*p* < 0.001). In the CA3 and DG sub‐regions, the levels of pS6 were significantly reduced in Cana‐treated males, but not in female mice (Figures 2c,d), with a significant interaction effect between treatment and sex (*p* < 0.001 for CA3 and *p* < 0.05 for DG). Thus, in the hippocampus, sex differences on pS6 were blunted by Cana treatment. The insulin‐stimulated pS6 correlated with increased pAkt in the hypothalamus and hippocampus of Cana‐treated male mice compared to control (Figures S3a,b,e,f,i, and j). In contrast, insulin‐stimulated pS6 levels were upregulated in female mice, regardless of treatment (Figures S3c,d,g,h,k, and l).

### 2.3 Cana treatment reduces the age‐associated inflammatory response in microglia and astrocytes

**FIGURE 3:**
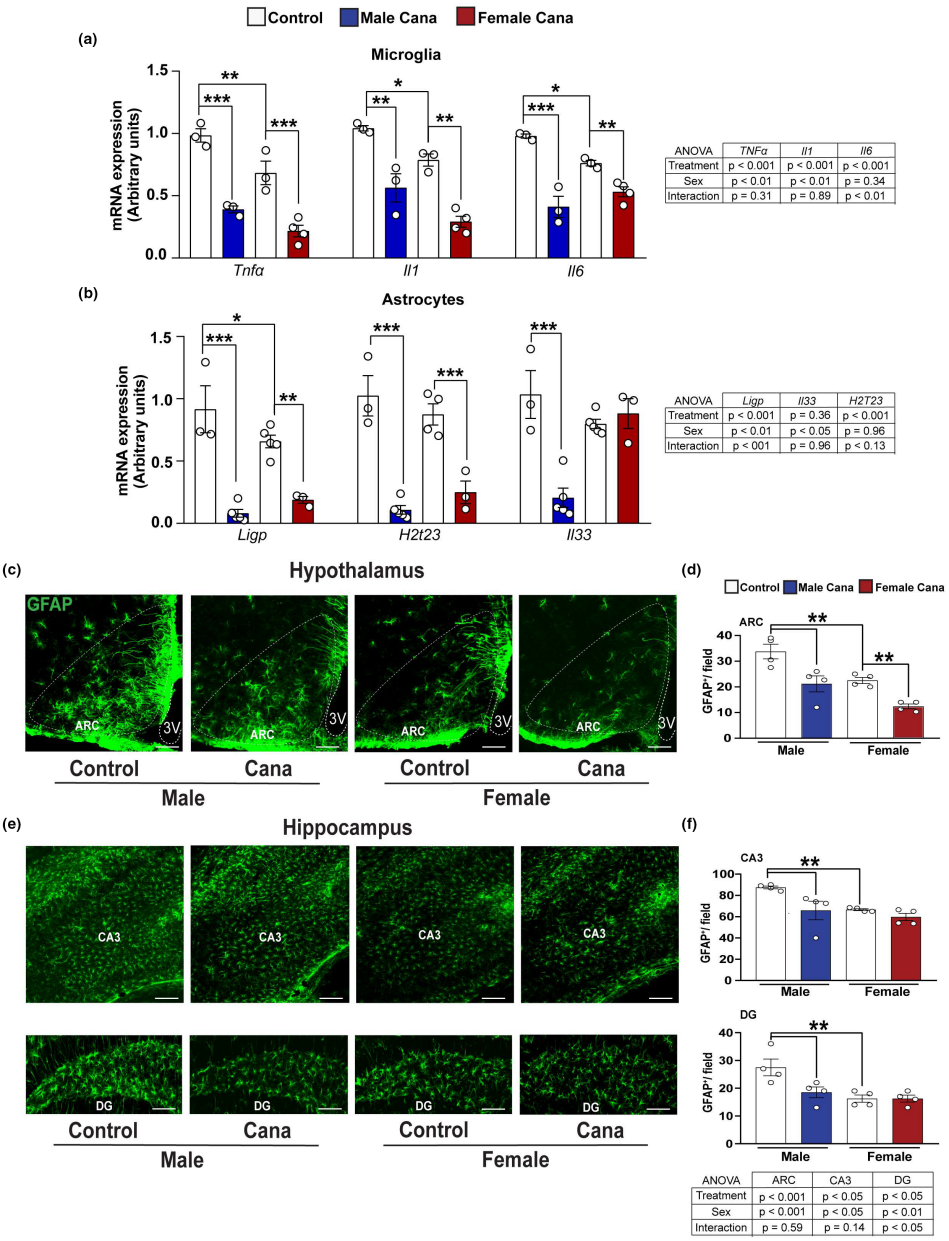
Neuroinflammation in aged Cana‐treated mice. Expression of *Tnfa*, *Il1*, and *Il6*, from isolated microglia (a) and *Ligp, H2t23, and Il33* from isolated astrocytes (b) of 30‐months‐old control and Cana‐treated male and female mice. Error bars show SEM for *n* = 3–5 mice/group. Data were analyzed by 2‐factor ANOVA and further analyzed with the Newman–Keuls post hoc test (*p < 0.05, **p < 0.01, ***p < 0.001). Brain sections of male and female mice were analyzed for hypothalamic and hippocampus GFAP^+^ astrocytes. Representative images showing immunostaining in the arcuate nucleus of the hypothalamus (ARC) (c), CA3, and dentate gyrus (DG) hippocampus areas (e) of 26–28 months‐old control and Cana‐treated mice. Scale bars: 200 μm. 3 V, third ventricle. Numbers of cells immunoreactive for GFAP in the ARC (d), CA3, and DG (f) from indicated male and female mice; error bars show SEM for *n* = 4 mice/group. Data were analyzed by 2‐factor ANOVA and further analyzed with the Newman–Keuls post hoc test (**p < 0.01). The tables demonstrate the two‐factor ANOVA analysis

Activation of microglia and astrocytes is fundamental for neuroinflammation, which in turn is tightly linked to insulin resistance in the aging brain (Komleva et al., 2020). We assessed whether Cana treatment modulated inflammatory cytokine production by microglia and astrocytes in the aging brain. Microglia and astrocytes were purified from 30‐month‐old mice by a density gradient, followed by cell sorting of whole brains (Figures S4a–c). Cells obtained by density gradient separation were then labeled with CD45/CD11b antibodies and sorted to obtain populations enriched for CD11b^+^/CD45^low^ cells, markers characteristics of resident brain microglia, and CD11b^+^/CD45^hi^ cells, which represent systemic macrophage populations. Astrocytes were sorted using the anti‐ACSA2 antibody, an astrocyte surface antigen (Kantzer et al., 2017) (Figures S4d,e). Microglia and astrocyte preparations were verified by the expression of microglia‐specific or astrocyte‐specific genes, respectively (Figures S4f,g). Cana treatment significantly reduced the expression of mRNA for the inflammatory cytokines *Tnfα*, *Il6*, and *Il1* in microglia in both male and female mice as compared to control microglia, with an interaction effect between Cana and sex only for *Il6* gene expression (*p* < 0.01) (Figure 3a). Further, Cana treatment significantly reduced the expression of the neuroinflammatory A1‐reactive genes, *Ligp* and *H2t23*, in astrocytes (Figure 3b). *Il33 is* a cytokine that mediates hippocampal synaptic plasticity and was shown to increase in astrocytes with age (Carlock et al., 2017) and (Wang et al., 2021). Interestingly, the expression of *Il33* by astrocytes was reduced by Cana treatment only in male but not in female mice (sex x treatment interaction *p* < 0.001) (Figure 3b).

### 2.4 Cana treatment reduces age‐associated microgliosis and astrogliosis in the region‐ and the sex‐specific manners

**FIGURE 4:**
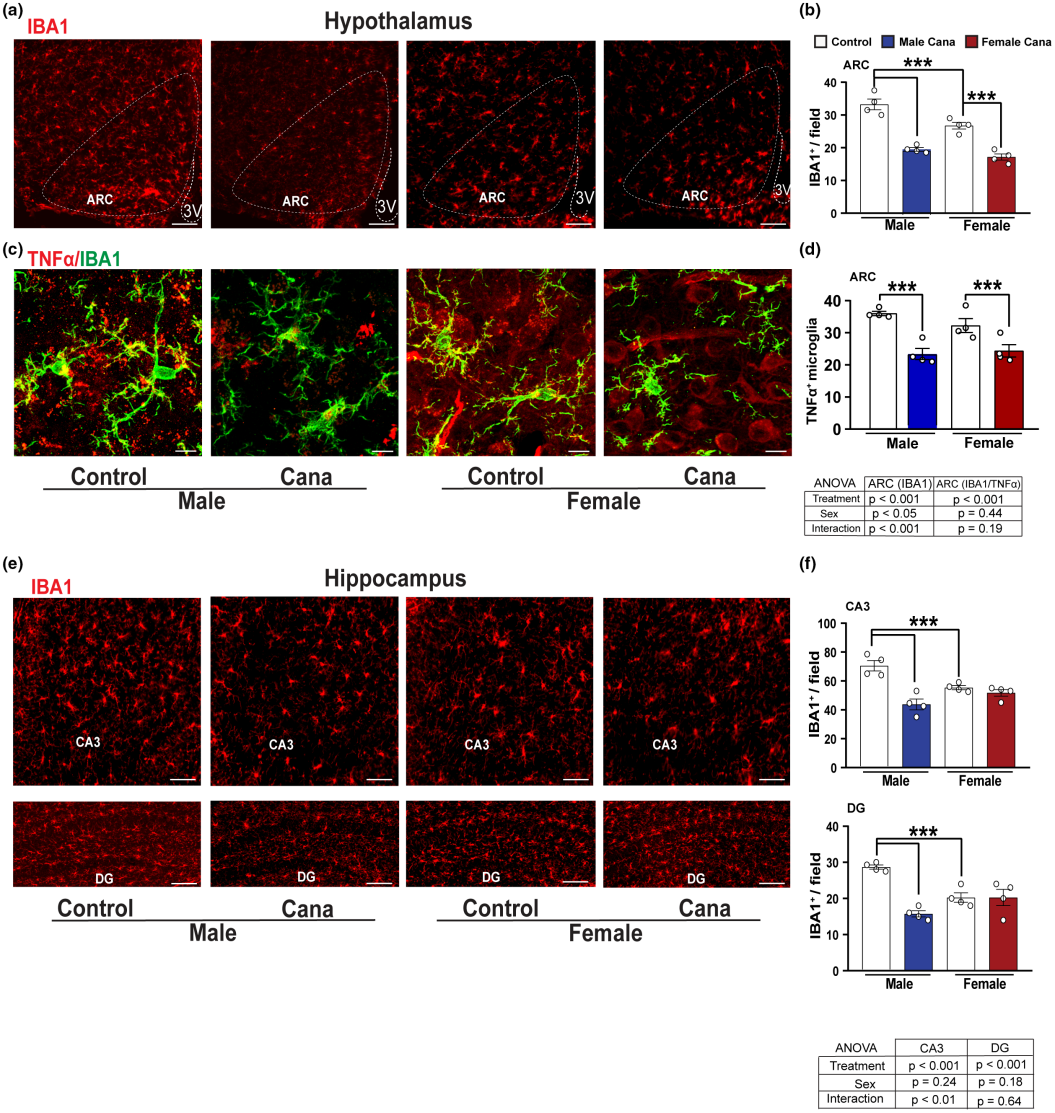
Microgliosis in aged Cana‐treated mice. Representative images showing Iba1^+^ cells (red) (a) and confocal images showing Iba1 (green) merged with TNF‐α (red) (c) immunostaining in the arcuate nucleus of the hypothalamus (ARC) of control and Cana‐treated 26–28 months‐old male and female mice. Numbers of cells immunoreactive for (b) Iba1 and (d) TNF‐α^+^/Iba1^+^ in the ARC of control and Cana‐treated male and female mice. (e) Representative images showing Iba1^+^ cells (red) in the CA3, and DG of 26–28 months‐old control and Cana treated mice. Numbers of cells immunoreactive for Iba1 in the CA3 and DG (f) from indicated male and female mice; error bars show SEM for *n* = 4 mice/group. Scale bars: 200 μm, 10 μm on merged images. 3 V, third ventricle. Data were analyzed by 2‐factor ANOVA and further analyzed with the Newman–Keuls post hoc test (***p < 0.001). The tables demonstrate the two‐factor ANOVA analysis

To assess the region‐ and sex‐specific effects of Cana on neuroinflammation we examined the activation state of astrocytes and microglia in aged brains. We evaluated the effect of Cana on astrogliosis in both the hypothalamus and hippocampus using glial fibrillary acidic protein (GFAP) as a marker for the astrocytes. In aged mice, we found about 1.5‐fold lower numbers of hypothalamic astrocytes in females (*p* < 0.001) as in our previous studies using younger mice, 12 or 22 months of age (Sadagurski et al., 2017). We similarly detected lower numbers of the astrocytes in the hippocampus of female mice as compared to males (*p* < 0.05). Cana treatment reduced astrogliosis in the hypothalamic ARC in both male and female mice (Figures 3c,d). In the hippocampus, Cana reduced astrogliosis in the sub‐regions CA3 and DG in males, but not in female mice (Figures 3e,f), with a significant interaction effect between drug (treatment) and sex in DG (*p* < 0.05). In addition, both male and female mice treated with Cana showed a significantly reduced number of hypothalamic microglia cells positive for the ionized calcium‐binding adapter molecule 1 (Iba1), a microglia‐specific marker (Figures 4a,b). The same effect was also observed by staining with TMEM119, another microglia marker (Figure S5). Hypothalamic Iba1^+^ cells produced tumor necrosis factor‐alpha (TNF‐α), indicating an inflammatory state (Figures 4c,d). Cana significantly reduced TNFα, production by microglia in both sexes in the ARC (*p* < 0.001). Similar to the hypothalamic astrogliosis, we found a significant effect of sex on hypothalamic microgliosis (*p* < 0.05), with lower microglia numbers in females compared to male mice. Sex effect was also observed in microglia numbers in the hippocampus (Figures 4e,f). Cana reduced microgliosis in sub‐regions CA3 and DG in male, but not in female mice (Figures 4e,f), with a significant interaction effect between drug and sex only in CA3 (Figure 4f, *p* < 0.01). Cana had no effect on microglial numbers in the female hippocampus in both CA3 and DG regions (Figures 4e,f). The total number of DAPI^+^ cells in the hippocampus, measured in CA1, CA2, CA3, and DG, was similar among groups (Figure S6a,b). We were not able to reliably detect TNFα by immunostaining in the hippocampus (data not shown).

**FIGURE 5:**
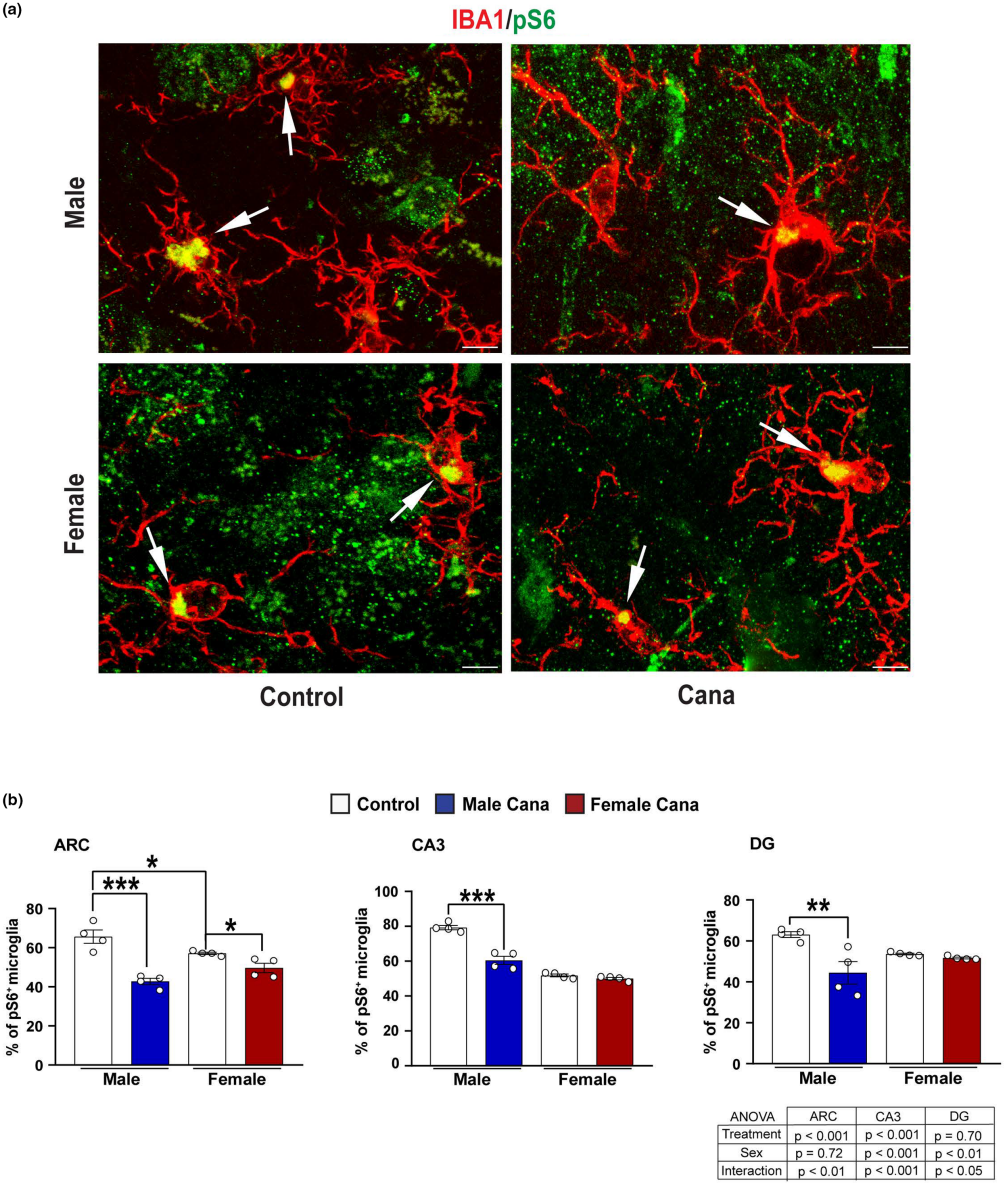
Microglial pS6 expression in Cana treated aged mice. (a) Representative confocal images of phosphorylated S6 (pS6, green) and Iba^+^ cells (red) in the arcuate nucleus of the hypothalamus (ARC) of 26–28 months‐old control and Cana treated male and female mice. Scale bars: 5 μm, 3 V, third ventricle. (b) Percentage of Iba1^+^ pS6 in the ARC, CA3, and DG of male and female mice. Error bars show SEM for *n* = 4 mice/group. Data were analyzed by 2‐factor ANOVA and further analyzed with the Newman–Keuls post hoc test (**p < 0.01; ***p < 0.001). The tables demonstrate the two‐factor ANOVA analysis. Images of lower magnification in the hypothalamus and hippocampus are shown in Figure S8

Cana levels in plasma and the hippocampus of female mice following exposure to Cana were measured using high‐performance liquid chromatography/mass spectrometry(MS)/MS (Mohamed et al., 2019). Plasma levels of Cana in treated female mice ranged from 4.38 to 6.03 μg/ml (*N* = 4), in accordance with the range reported for humans administered this drug (Devineni & Polidori 2015). Cana levels in the hippocampus of treated female mice ranged from 0.56 to 0.46 ng/mg (*N* = 4) and were correlated with previously measured Cana levels from the whole brain of female mice (Miller et al., 2020). Interestingly, we detected a significant effect of sex (*p* < 0.001) in *Sglt2* expression levels, with females having lower *Sglt2* levels in the hypothalamus, but significantly higher *Sglt2* expression levels in the hippocampus than males (Figure S7). Thus, the absence of the Cana effect on females cannot be attributed to reduced expression levels of *Sglt2* or Cana levels in the hippocampus.

Overall, these data indicate that sex‐specific and region‐specific effects of Cana supplementation on neuroinflammation in brain areas are critical for the integration of metabolic homeostasis and memory formation.

### 2.5 Cana treatment reduces mTOR signaling in aged microglia in a sex‐specific manner

Microglia from aged mice demonstrated upregulation of mTOR‐downstream signaling and the production of inflammatory cytokines (Keane et al., 2021). Interestingly, we detected sex‐specific differences in pS6 in aged microglia, with females exhibiting lower pS6 levels in both hypothalamus (*p* < 0.05) and hippocampus (p < 0.001 for CA3). Reduced pS6 was detected in hypothalamic microglial cells in response to Cana treatment in both male and female mice with a stronger effect in male mice (Figure 5a,b and Figure S8). The hippocampus, however, is different: the fraction of pS6 positive microglia cells was significantly reduced in the hippocampus in response to Cana only in male mice (for an interaction effect between treatment and sex *p* < 0.0001 for CA3 and *p* < 0.05 for DG) (Figures 5a,b). This sexually dimorphic effect of Cana on pS6 in microglia is consistent with the male‐specific lifespan extension observed in Cana‐treated animals (Miller et al., 2020).

### 2.6 Cana treatment improves locomotor activity and overall behavior function in aging

**FIGURE 6:**
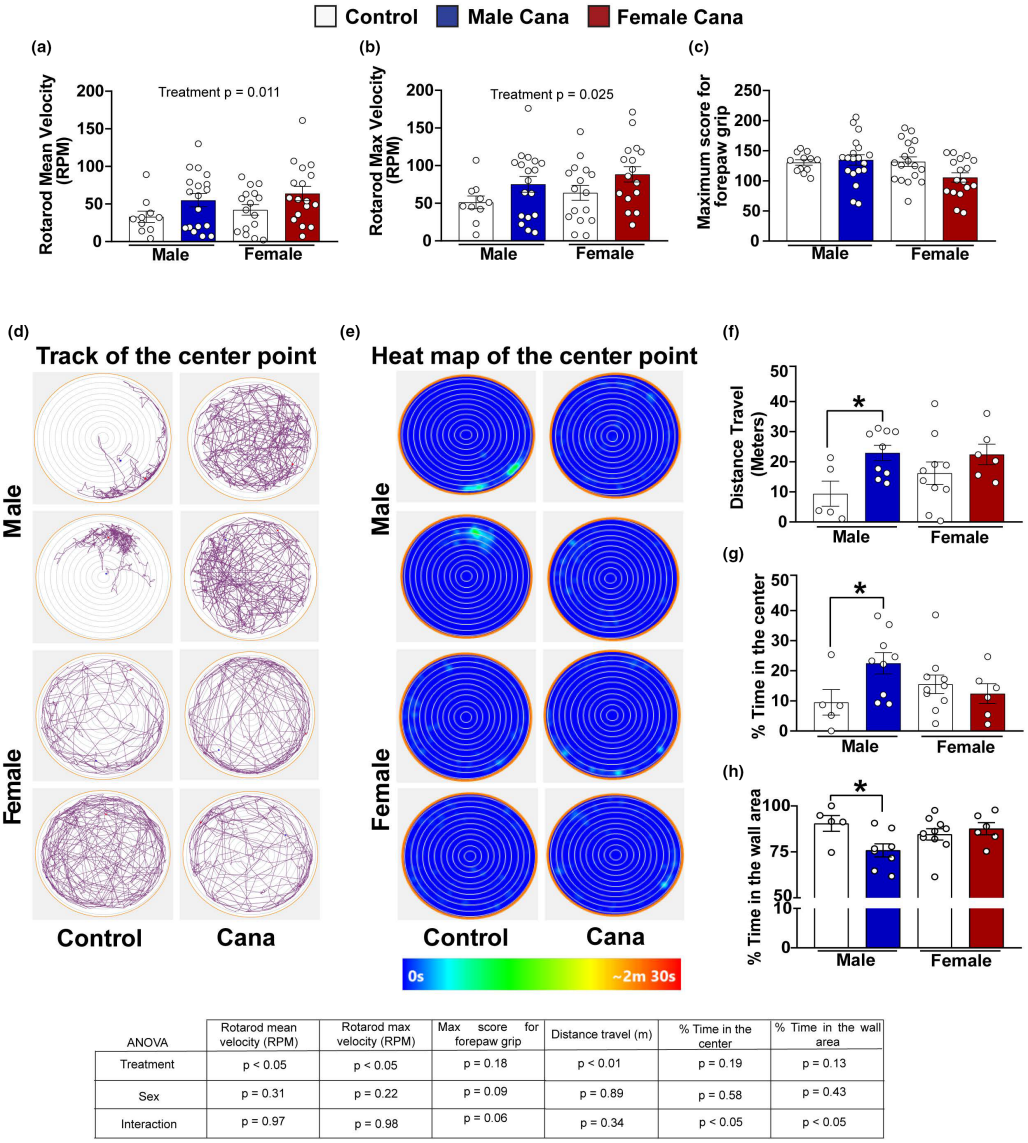
Neuromuscular function, locomotor activity, and exploratory behavior in Cana treated aged mice. (a) Rotarod means velocity (RPM), (b) maximum velocity, and (c) maximum score for forepaw grip of 22‐month‐old control and Cana treated male and female mice. Error bars show SEM for *n* = 12–19 mice/group. Representative images from the open field test (OFM) of 30 months‐old control and Cana‐treated male and female mice. (d) Track of the center point represents the total distance traveled by the mice during 10 min; (e) Heat map of the center point, the light‐blue color represents the time spent during 10 min in the same spot. (f) Total distance traveled (meters) in the OFM. (g) Percentage time spent in the center of OFM.(h) Percentage time spent in the outer wall area. Error bars show SEM for *n* = 5–9 mice/group. Two‐factor ANOVA analyzed data and further analyzed with the Newman–Keuls post hoc test (*p < 0.05). Tables show the two‐factor ANOVA analysis

To assess neuromuscular activity, we assessed rotarod performance at 22 months of age using an acceleration protocol where mice were tested for their ability to balance on a progressively accelerating rotarod (Garratt et al., 2019). The ability of mice to maintain balance on the rod declines with age, while Cana treatment significantly improves balance ability and was associated with a longer time on the accelerated rotarod (mean of 3 trials) before fall (*p* = 0.011) independent of sex (Figure 6a). Similarly, the Cana effect on the maximum performance on the rotarod was increased (*p* = 0.025), while no interaction with sex was detected (Figure 6b). Next, we assessed grip strength. Forepaw grip scores did not differ significantly between Cana‐treated or untreated mice at this age (Figure 6c). Body weight was significantly decreased approximately by 9 and 7 g in Cana‐treated female and male mice, respectively (45.92 ± 1.86 for male control vs. 39.05 ± 1.14 for male Cana; 43.56 ± 1.92 for female control vs. 34.65 ± 1.08 for female Cana), indicating that improved rotarod performance may be due to, at least in part, lower body weight in the Cana groups at this age. To evaluate general activity levels, gross locomotor activity, and exploratory behavior, we performed an open field test in 30‐month‐old control and Cana‐treated mice. Cana‐treated male mice were significantly more active than control males as shown by the increased total distance traveled (Figures 6d,e,and f). The latency to enter the center of the arena and the numbers of visits and duration in the center were significantly increased in males as well (Figure 6g). There was a significant interaction between drugs and sex (*p* < 0.05). A significantly increased duration in the center of the arena of Cana treated male mice, compared to control males, suggested reduced anxiety‐related behavior, and increased willingness to explore a new environment (Shoji et al., 2016; Gerasimenko et al., 2020). None of these measures was altered in Cana‐treated female mice (Figures 6d‐h).

## 3 DISCUSSION

We identified a broad spectrum of cellular and functional changes that were improved by Cana treatment in genetically heterogeneous aged mice. Cana treatment significantly improved central insulin sensitivity, reduced age‐associated gliosis, and decreased microglial pS6 levels in a sex‐specific manner, predominantly benefiting old male, but not female mice. Measures of physical performance were significantly improved by Cana in male but not in female mice. We posit that Cana, and potentially other SGLT2 inhibitors, protect against age‐associated neurological, behavioral, and metabolic dysfunction, even in the absence of diabetes.

Cana treatment improved central insulin sensitivity in the hypothalamus and hippocampus of aged UM‐HET3 male mice, while females maintained adequate insulin signaling regardless of treatment. In our previous study, we showed that Cana levels in brain extracts and plasma were significantly higher in females than in male mice (Miller et al., 2020). Thus, Cana‐induced brain insulin sensitivity in males cannot be attributed to the higher systemic or central levels of the drug. A recent clinical study with the SGLT2i empagliflozin reported improved hypothalamic insulin sensitivity in subjects with prediabetes and obesity (Kullmann et al., 2021), although the study was not randomized in a sex‐stratified manner. The mechanisms for improved central insulin sensitivity are still not fully clear. However, these authors speculated that SGLT2i can modulate the activity of the autonomic nervous system with a shift from sympathetic to parasympathetic tone (DeFronzo et al., 2017; Kullmann et al., 2021). The current data, showing Cana‐induced improvement in central insulin signaling in male mice, implies beneficial effects of Cana beyond those related to systemic glucose control per se. Indeed, insulin resistance in the brain has been associated with acceleration of the aged‐induced cognitive decline in animal models and humans (Freude, Schilbach, et al., 2009; Liu et al., 2011; Talbot et al., 2012). Sex‐specific effects in brain insulin sensitivity may underlie or partially contribute to the benefit of Cana on lifespan in males.

The importance of central insulin signaling for cognitive health in humans has encouraged trials of nasal insulin delivery to slow the progression of AD (Claxton et al., 2015; Salameh et al., 2015; Arnold et al., 2018). Regardless, gene knockout studies have demonstrated that reducing insulin signaling in the central nervous system (CNS) benefits lifespan and cognitive function (Selman et al., 2008; Freude, Hettich, et al., 2009; Killick et al., 2009) (Schubert et al., 2003; Taguchi et al., 2007; Sadagurski et al., 2011). For example, knockout of neuronal Irs2 early in brain development extends lifespan and improves hippocampal function, despite resulting in weight gain and insulin resistance (Taguchi et al., 2007). How peripheral insulin resistance and central insulin signaling modulate hippocampal function during aging is an important but unsolved clinical question that requires more studies to differentiate the effects of central from peripheral insulin resistance (Kullmann et al., 2016; Arnold et al., 2018).

Neuroinflammation, driven by the activation of glial cells, increases with age in mouse and human brains and is associated with decreased central insulin signaling (Sierra et al., 2007; Limbad et al., 2020). Interaction between inflammation and insulin sensitivity is well documented for many tissues and is beginning to be appreciated in the CNS as well (Shoelson et al., 2006; Milstein & Ferris 2021). We show that Cana treatment significantly reduced microglia and astrocytes activation in sex‐ and region‐specific manners. In aged male mice treated with Cana, we found reduced microgliosis and astrogliosis in both the hypothalamus and hippocampus, while in female mice we found reductions in hypothalamic neuroinflammation only. This was evidenced by significantly reduced expression of the pro‐inflammatory cytokines in microglia with Cana treatment. Age‐related increases in pro‐inflammatory cytokines in the brain are thought to be detrimental to humans or rodents and correlate with deficits in cognitive function (Barrientos et al., 2015). Aged‐induced microgliosis promotes activation of A1‐like reactive astrocytes that in turn upregulate many mediators with neurotoxic function, causing neuronal death and neurodegeneration (Liddelow et al., 2017; Clarke et al., 2018). Ample evidence suggests that impaired peripheral metabolism associated with hypothalamic inflammation contributes to impairments in insulin sensitivity (Jais & Bruning 2017). Our previous study revealed improved peripheral glucose tolerance and lower body weight in aged male and female mice with Cana treatment (Miller et al., 2020). In the current study, we show that Cana reduced hypothalamic gliosis in both sexes, which may contribute to improved whole‐body metabolism. Reduced hippocampal gliosis in Cana‐treated male, but not female mice, is consistent with the sex‐specific lifespan increase seen in males and might be among the principal mechanisms by which Cana can improve cognitive function in aging.

Among commonly used SGLT2i, Cana has the least specificity and can potentially inhibit both SGLT1 and SGLT2 transporters (Ohgaki et al., 2016). SGLT1 and SGLT2 have been detected in many areas of the brain (Głuchowska et al., 2021). Brain expression of SGLT2 is lower than SGLT1 but was detected in the microvessels of the blood–brain barrier, hippocampus pyramidal and granular cells, and astrocytes in the ventromedial hypothalamus (Poppe et al., 1997; Enerson & Drewes 2006; Fan et al., 2015; Tahara et al., 2016; Koepsell 2020; Nguyen et al., 2020). Thus, while Cana's effects on CNS can be indirect, secondary to peripheral changes, or mediated via the activity of autonomic inputs to the hypothalamus (Spallone & Valensi 2021), it is plausible that Cana can attenuate hypothalamic gliosis directly via binding to the SGLT1 or SGLT2. Additional studies will be required to address this question.

Cana treatment was associated with reduced pS6 in the hippocampus of 30‐month‐old male mice, implicating reduced mTOR signaling. Previous studies have shown that Cana's effects on metabolic health were associated with suppressed mTOR signaling, as shown by reduced hepatic pS6 signaling accompanied by increasing AMPK activity in young animals (Osataphan et al., 2019). That study did not, however, evaluate the effects of Cana treatment in female mice. The age trajectory of mTORC1 (mTOR complex 1) and mTORC2 (mTOR complex 2) activity varies by tissue, strain, sex, and feeding status (Baar et al., 2016). Microglia from aged mice upregulated mTOR signaling, affecting targets that regulate translation, including 4EBP1 and pS6 (Keane et al., 2021). We show that pS6 is significantly reduced (~25%) in microglia in response to Cana treatment in the hypothalamus and hippocampus of male mice. However, in female mice reduced pS6, although significant, was detected only in the hypothalamus and reached (~8%). While not directly tested in this study, Cana‐mediated beneficial effects on aging brain phenotypes may be associated with decreased mTOR signaling.

Cana treatment was associated with improvements in locomotor activity (rotarod), and overall behavior function (open field test). These effects were seen in Cana‐treated males, but not in female mice. Previous studies reported deterioration in a battery of behavioral tests in C57BL/6 mice from 2 to 12 months of age (Shoji et al., 2016). In the current study, we show that Cana treatment of the genetically diverse UM‐HET3 mice significantly improved rotarod performance in male mice, but did not affect grip strength. Additionally, we found that the distance traveled for Cana‐treated male mice in the open field test significantly increased, suggesting improved overall activity levels at 30 months of age. Accordingly, Cana‐treated aged male mice spent significantly less time in the wall area and more at the center of the maze suggesting the willingness to explore a new environment (Seibenhener & Wooten 2015; Shoji et al., 2016; Feyissa et al., 2017; Perals Bertomeu et al., 2017). We cannot exclude the possibility that the behavior changes observed in Cana‐treated male mice resulted from body weight changes. Cana males are lighter than controls at ages 12 and 18 months, however, when body composition was measured by 22 months of age there was no significant difference in total mass, lean mass, or fat mass between control and Cana males. On the other hand, Cana led to a significant decline in total mass and in fat mass in females at that age (Miller et al., 2020). Lean mice on caloric restriction demonstrate preserved memory, learning, and reduced anxiety with aging (Parikh et al., 2016), while obese mice exhibit cognitive deficits, anxiety, and depressive‐like behaviors (Almeida‐Suhett et al., 2017). However, we did not observe beneficial effects of Cana on female activity and exploratory behaviors, suggesting that male‐specific improvements in activity and overall behavior function are not necessarily attributable to weight changes. Our results imply that Cana can be beneficial for delaying frailty and may affect more than one system.

In summary, we show that Cana, with recently identified anti‐aging properties, can alleviate age‐related neuroinflammation, improve central insulin sensitivity and increase overall physical activity. Our findings are in line with other recent clinical studies and rodent work, indicating that Cana and other SGLT2i can improve kidney, and heart function, and delay oncogenesis. Our data suggest possible new applications for Cana that require wide‐ranging evaluation of patients already receiving this FDA‐approved drug for specific indications.

## 4 MATERIALS AND METHODS

### 4.1 Animals

Procedures involved in this study were approved by the University of Michigan Committee on the Use and Care of Animals. All the animals were fed Purina 5LG6 until 7 months of age. Animals were maintained under temperature and light‐controlled conditions.

### 4.2 Experimental diets

Cana was purchased from Steraloids, Inc. (Newport, RI, USA) and mixed at a dose of 14.4 milligrams per kilogram diet (14.4 ppm). Animals of 7 months of age were randomly categorized into control or Cana treatment. Control animals were continuously fed with 5LG6, and the treatment group was fed with the Cana diet continuously from 7 months of age.

### 4.3 Perfusion and immunolabeling

Mice were anesthetized and perfused using phosphate buffer saline (PBS) (pH 7.5) followed by 4% paraformaldehyde. Brains were postfixed, dehydrated, and then sectioned coronally (30 μm) using a sliding microtome, followed by immunofluorescent analysis as described (Sadagurski et al., 2017). For immunohistochemistry brain sections were washed with PBS six times, blocked with 0.3% Triton X‐100 and 3% normal donkey serum in PBS for 2 h; then the staining was carried out with the following primary antibodies overnight: rabbit anti‐GFAP (1:1000; Millipore, Cat. No. ab5804), rabbit anti FoxO1 (1:200; Cell signaling Cat. No. ab2880s), and mouse anti‐NeuN (1:1000 Cell signaling Cat. No. MAB377). For goat anti‐Iba1 (1:1000 Abcam Cat. No. ab5076) and rabbit anti‐TMEM (1:300; Abcam. Cat.No.ab209064), rabbit anti pS6 (1:100; Cell signaling Cat. No. 2215) immunostaining brain sections were pretreated with 0.5% NaOH and 0.5% H_2_O_2_ in PBS for 20 min. After the primary antibody brain sections were incubated with AlexaFluor‐conjugated secondary antibodies for 2 h (Invitrogen). Microscopic images of the stained sections were obtained using an Olympus FluoView 500 and Laser Scanning Confocal Microscope Zeiss LSM 800.

### 4.4 Quantification

For the cell quantification and immunoreactivity analysis, images were taken from at least 3 sections containing the hypothalamus and hippocampus for each brain between bregma −0.82 and −2.4 mm (according to the Franklin mouse brain atlas). Serial brain sections were made at 30 μm thickness. All sections were arranged from rostral to caudal to assess the distribution of labeled cells from the equivalent sections for all stains. Fiji‐ImageJ was used to count GFAP and Iba1 positive cells and to measure immunoreactivity. The maximum and minimum thresholds were set up equally for all the images. For FoxO1 localization analysis, confocal images of FoxO1, NeuN, and DAPI were taken using the multiphoton laser‐scanning microscope (LSM 800, ZEISS) equipped with a 63X objective. The images were taken in the ARC, DG, and CA3 regions (3–4 images/mouse). Stacks of consecutive images taken at 0.35‐μm intervals were sequentially acquired, and 30 optical sectioning of the optical axis (*z*‐axis) were reconstructed to 3D using Fiji‐Image J. All microscopy images and quantifications were performed by investigators that were blinded to the sample's ID.

### 4.5 Cell sorting

Microglia and astrocytes were isolated by density gradient from control and Cana‐treated mice. This was followed by cell sorting via staining with CD45/CD11b to identify microglia (CD11b^+^/CD45^low^), characteristics of resident brain microglia, and distinguish them from CD45^hi^ systemic macrophage populations. Astrocytes were sorted by ACSA2 (Kantzer et al., 2017). To validate the sorting technique we performed the qPCR with sorted microglia and astrocytes using GFAP (astrocytes specific marker) and TMEM119 (microglia specific marker).

### 4.6 Quantitative Real‐Time PCR

Total RNA was isolated from FACS sorted microglia and astrocytes using Trizol reagent (Invitrogen, #15596026). The concentration of 1000 ng of RNA was used for cDNA synthesis using a High Capacity cDNA Reverse Transcription Kit (BioRad, #1708891). More details are provided in Supplementary Methods.

### 4.7 Rotarod and grip strength tests

Animals were tested for their ability to balance on an accelerating rotarod at 22 months of age. Animals were placed on the rotarod and the trial began with the spindle revolving at 5 revolutions per minute (RPM) and increased to 40 RPM gradually over a 5‐min period. The time at which the animal fell off the rotarod was used as a score, with each animal tested three times and the mean score used in the analysis. Animals were also tested for grip strength using an EB1‐BIO‐GT3 grip strength meter with an EB1‐GRIP‐Mouse Grid. Subjects were removed from their cage by the base of the tail and suspended above the grid until their forepaws griped the grid. The tail was gently pulled in a horizontal direction away from the grid until the mouse released its grip. The maximal force was recorded. Each animal was tested six times with a 10 s rest between each. The mean of the six tests was used for analysis. All tests were conducted by an experimenter blind to the treatment group.

### 4.8 Open field maze

The open‐field arena was 23 cm in radius and made with polyvinyl chloride. The center of the arena was defined as a 10 cm radius circle from the center and the outside of this center circle is defined as the outer area (wall area) of the arena. Testing was conducted under bright light. Mice were tested for 10 min inside the arena. At the beginning of the test, animals were placed in the center of the open field arena and the movement of animals was recorded with a Nikon camera. Arenas were cleaned with 70% isopropyl alcohol between trials and the observer was in a different room to the arena throughout the testing time. After recording, the videos were analyzed using Any‐Maze Video Tracking System^©^ v 6.34 to analyze the distance moved, the percent time spent in the center of the open field arena, and the percent time spent in the wall area of the arena in each trial. Using Any‐Maze Video Tracking System^©^ v 6.34 a series of 20, 40, 60, 80, 100, 120, 140, 160, 180, 200, and 220 mm radius zones were identified and used to evaluate the animal track and collect the data for the locomotor activity. The six center circular zones were identified as the inner zone and the other six circular zones were identified as the outer zone.

### 4.9 Statistical analysis

Data sets were analyzed using a two‐factor analysis of variance (two‐way ANOVA), using the general linear model function and a full factorial model, which included an effect of treatment, the effect of sex, and the interaction effect between sex and treatment followed by Newman–Keuls post hoc test. All data were presented as mean ± SEM. *p* < 0.05 was considered significant. TIBCO Statistica® v. 13.5.0.17 was used for statistical analysis.

## AUTHOR CONTRIBUTIONS

LD, JJ, and HJ carried out the research. RAM provided drug‐treated and control mice, and reviewed and revised the manuscript. MS designed the study, analyzed the data, wrote the manuscript, and is responsible for the integrity of this work. All authors approved the final version of the manuscript.

## CONFLICT OF INTEREST

No conflicts of interest are declared by the authors.

## Supporting information


Appendix S1

## Data Availability

The data that support the findings of this study are available from the corresponding author upon reasonable request.
