# Supplementary material for: Neuroprotective effects of Canagliflozin: Lessons from aged genetically diverse UM‐HET3 mice
Source: Aging Cell. 2022 Jun 15;21(7):e13653. doi: 10.1111/acel.13653 (PMC9282842; doi:10.1111/acel.13653)

SUPPLEMENTARY MATERIAL

Neuroprotective effects of Canagliflozin: lessons from aged genetically diverse UM-HET3 mice

[1]Hashan S. M. Jayarathne, [1]Lucas K. Debarba, [1]Jacob J. Jaboro, [2]Brett C. Ginsburg

[3]Richard A. Miller, *[1]Marianna Sadagurski

[1]Department of Biological Sciences, IBio (Integrative Biosciences Center), Wayne State University, Detroit, Michigan, MI, [2]Department of Psychiatry and Behavioral Sciences, University of Texas Health Science Center, San Antonio, TX
[3]Department of Pathology and Geriatrics Center, University of Michigan, Ann Arbor, MI

Running title: Cana effect on aging brain

Keywords: Canagliflozin, brain, hypothalamus, hippocampus, metabolism, longevity

Corresponding author:

Marianna Sadagurski,
Department of Biological Sciences,
Integrative Biosciences Center
Wayne State University
6135 Woodward, Detroit, MI 48202
Phone: (313) 577 8637
Email: sadagurski@wayne.edu

Supplementary Materials and Methods

Measurement of Canagliflozin in mouse brain and plasma using HPLC/MS/MS

Analyses of Cana in mouse brain and plasma were conducted using a modification of a published method (Mohamed *et al.* 2019). The HPLC/MS/MS system consisted of a Shimadzu SIL 20A HT autosampler, LC-20AD pumps (2), and an AB Sciex API 4000 tandem mass spectrometer, with turbo ion spray. The LC analytical column was an ACE Excel C18-PFP (75 x 3.0 mm, 3 microns) purchased from Mac-Mod Analytical (Chaddsford, PA) and was maintained at 25°C during the chromatographic runs using a Shimadzu CT-20A column oven. Mobile phase A contained 0.1% formic acid dissolved in water. Mobile phase B contained 0.1% formic acid dissolved in 100% HPLC grade acetonitrile. The flow rate of the mobile phase was 0.4 ml/min. Canagliflozin and Lidocaine D10 (an internal standard obtained from Sigma Aldrich, St. Lous, MO) were eluted with a gradient. The initial mobile phase was 20% B and at 1 minute after injection was ramped to 100% B. From 4.0 min to 8.0 min the mobile phase was maintained at 100% B and 8.01 minutes was switched immediately back to 20% B and ran for 1.99 minutes to equilibrate the column before the next injection. The Cana transition was detected in positive mode at 445.3/267.2 Da. The internal standard, Lidocaine D10, transition was detected at 245/96 Da.

Mouse plasma: calibrator samples were prepared daily by spiking blank plasma with Cana Apex BioScience (Glen Allen, VA) to achieve final concentrations of 0, 10, 50, 100, 500, 1000, 2500, 5000, and 10000 ng/ml. Calibrator and unknown plasma samples (0.05 mL) were mixed with 10 µL of 1 µg/mL lidocaine D10 and 1 ml of acetonitrile. The samples were vortexed vigorously and then centrifuged at 13,000 g for 5 min at 25°C. The supernatants were transferred to 5 ml polypropylene tubes and 3 ml of ethyl acetate was added. The samples were shaken at low speed for 10 min and then centrifuged at 3,724 g for 10 min at 10°C. The clear supernatants were transferred to a new 5 ml tube and dried to residue under a warm nitrogen stream. The residue was then redissolved in 50 µL of mobile phase B, vortexing for 30 seconds. The samples were

transferred to injection vials and 10 µL was injected into the HPLC/MS/MS. The ratios of Cana peak areas to lidocaine D10 peak areas for each unknown sample were compared against a linear regression of the ratios obtained by the calibration samples to quantify Cana. The concentration of Cana was expressed as µg/mL plasma.

Mouse hippocampus: each hippocampal section (39 ± 7 mg, mean ± SD) was weighed in a polypropylene tube and then a 10x volume of 75% methanol was added. Each sample was thoroughly homogenized. Calibrator samples were prepared by spiking a control brain homogenate to achieve final concentrations of 0, 10, 20, 40, 80, 100, and 400 ng/ml. Briefly, 0.2 ml of the calibrator and unknown brain homogenates were mixed with 10 µL of 1 µg/mL lidocaine D10. The samples were vortexed vigorously and then centrifuged at 13,000 g for 5 min at 25ºC. The supernatants were transferred to clean 1.5 mL microcentrifuge tubes and centrifuged at 13,000 g for 2 min at 25oC. The samples were transferred to injection vials and 10 µL was injected into the HPLC/MS/MS. The concentration of Cana was expressed as ng/mg brain.

Quantitative Real-Time PCR

To detect the contaminated DNA we used the samples processed without the transcriptase reverse enzyme as negative controls. Quantitative real-time PCR was performed using the Applied Biosystems 7500 Real-Time PCR System (see supplementary table 1 for primers sequences). Each PCR reaction was performed in duplicate. As negative controls, we used water instead of the cDNA, and ß-actin was measured in each cDNA sample as the housekeeping gene. The ΔΔCT method was used to determine the gene transcripts in each sample. For each sample, the threshold cycle (CT) was measured and normalized to the average of the housekeeping gene (ΔCT = CT gene of interest - CT housekeeping gene). The fold change of mRNA in the rest of the samples relative to the control male group was determined by 2-ΔΔCT, where ΔΔCT = ΔCT other animal groups (Cana males, control females, Cana females) - ΔCT control male group. Data are shown as mRNA expression levels relative to the control males

Supplementary Legends

Supplementary Figure 1. Insulin→FoxO1 signaling in aged Cana-treated mice. Representative confocal images from the hypothalamus for FoxO1 (red) and DAPI (blue) in 30-months-old control and Cana-treated males stimulated with insulin (3 U/kg i.p.; 15min). Scale bars represent 200 µm (for split and merged channels) or 10 µm (on the left) showing merged cells of the indicated groups for cytoplasmatic (C) and nuclear (N) localization.

Supplementary Figure 2. pAKT expression in aged Cana-treated mice. Immunofluorescence for pAkt (red) and DAPI (blue) in 30-months-old control and Cana treated mice injected with insulin (3 U/kg i.p.; 15min) or saline. Representative images from the hypothalamus in males (A) and females (C), or hippocampus CA3 in males (E) and females (G), and dentate gyrus (DG) in males (I) and females (K) of control and Cana-treated mice are shown. Scale bars represent 200 µm showing merged images of the indicated groups. Quantification of positive cells in the ARC, CA3, and DG in males (B, F, J) and females (D, H, L). Gene expression for Irs1, Irs2, and Akt in the hypothalamus of male and female control and Cana-treated mice (M); error bars show SEM for n = 4-7 mice/group. Data were analyzed by 2-factor ANOVA and further analyzed with the Newman–Keuls post hoc test (***$p<0.001$). The tables demonstrate the two-factor ANOVA analysis.

Supplementary Figure 3. pS6 expression in aged Cana-treated mice. Immunofluorescence for pS6 (green) in 30-months-old control and Cana treated mice injected with insulin (3 U/kg i.p.; 15min) or saline. Representative images from the hypothalamus in males (A) and females (C), or hippocampus CA3 in males (E) and females (G), and dentate gyrus (DG) in males (I) and females (K) of control and Cana-treated mice are shown. Scale bars represent 200 µm. Quantification of positive cells in the ARC, CA3, and DG in males (B, F, J) and females (D, H, L); error bars show SEM for n = 4-7 mice/group. Data were analyzed by 2-factor ANOVA and further analyzed with

the Newman–Keuls post hoc test (***$p<0.001$). The tables demonstrate the two-factor ANOVA analysis.

Supplementary Figure 4. Representative plots of Fluorescence Activated Cell Sorting of cells and gene expression from aged Cana-treated mice. (A) Represents cells and (B) represents singlets that were sorted based on cell size, as determined by the area (A) and the height (H) of Light Forward Scatter (FSC), and cellular complexity/granularity, based on Light Side Scatter (SSC). Each dot represents one event (either a cell or debris) that has passed through the cytometer's detector. (C) Represents percent of live cells (Zombie dye). (D) Cells were then sorted based on CD11b and CD45 fluorescent intensity to identify microglia ($CD11b^{+}/CD45^{low}$). (E) Astrocytes were gated based on ACSA expression. Positive and negative gates were drawn based on the intensity of an unstained control sample. Gene expression of microglia-specific, *Tmem119* (F) and astrocyte-specific, *Gfap* (G). Error bars show SEM for n = 3-4 mice/group. Student's t-test (*$p < 0.05$, **$p < 0.01$).

Supplementary Figure 5. Co-localization of microglial markers Iba1 and TMEM119 in the hypothalamus and hippocampus of Cana-treated mice. Representative images of Iba1 (red) and TMEM119 (green) in the arcuate nucleus of the hypothalamus (ARC) and hippocampal CA3 and dentate gyrus (DG) of 26-28 months-old control and Cana treated mice. Scale bars: 200 μm, 3V, third ventricle.

Supplementary Figure 6. Nuclei staining in hippocampus of aged Cana-treated mice. (A) Representative images of nuclei staining (DAPI) of the hippocampus. (B) Immunoreactivity of DAPI in the Dentate Gyrus (DG), CA1, CA2, and CA3 regions of the hippocampus (4x) of 30-month-old male and female mice. Scale bars: 200μm. Error bars show SEM for n = 3 mice/group.

Supplementary Figure 7. *Sglt2* gene expression. *Sglt2* expression in the hypothalamus and hippocampus of 26-28-months-old control and Cana-treated male and female mice n = 4-5

mice/group. Data were analyzed by 2-factor ANOVA and further analyzed with the Newman–Keuls post hoc test. Effect of sex $p<0.001$.

Supplementary Figure 8. pS6 expression in Cana-treated mice. Representative images of phosphorylated S6 (pS6, green) expression and Iba^{+} cells (red) in the arcuate nucleus of the hypothalamus (ARC) (20x) and hippocampal CA3 and dentate gyrus (DG) (40x) of 26-28-months-old male and female control and Cana-treated mice. Arrows indicate colocalization. Scale bars: 200µm, 3V, third ventricle.

Supplementary Table 1: qPCR primer list

Target Gene	Forward 5'-3'	Reverse 5'-3'
B actin	AGCCATGTACGTAGCCATCCA	TCTCCGGAGTCCATCACAATG
Akt	GTGTCCAGTGTAGAATGACTC	ATCTGTCGGAGAACACACATG
Gfap	GTACCAGGACCTGCTCAAT	CAACTATCCTGCTTCTGCTC
Tmem119	GTGTCTAACAGGCCCCAGAA	AGCCACGTGGTATCAAGGAG
Tnfα	CCCTCACACTCAGATCATCTTCT	GCTACGACGTGGGCTACAG
Il1	CACAGCAGCACATCAACAAG	GTGCTCATGTCCTCATCCTG
Il6	CTCTGGGAAATCGTGGAAAT	CCAGTTTGGTAGCATCCATC
Il33	TGAGACTCCGTTCTGGCCTC	CTCTTCATGCTTGGTACCCGAT
Irs1	TTTGAAGACCATAACCCACCAC	ATTACACCAGTTCGTCCCTTTC
Irs2	TCCAGAACGGCCTCAACTAT	AGTGATGGGACAGGAAGTCG
Ligp1	GGGGCAATAGCTCATTGGTA	ACCTCGAAGACATCCCCTTT
H2-t23	GGACCGCGAATGACATAGC	GCACCTCAGGGTGACTTCAT

Supplementary Figure 1

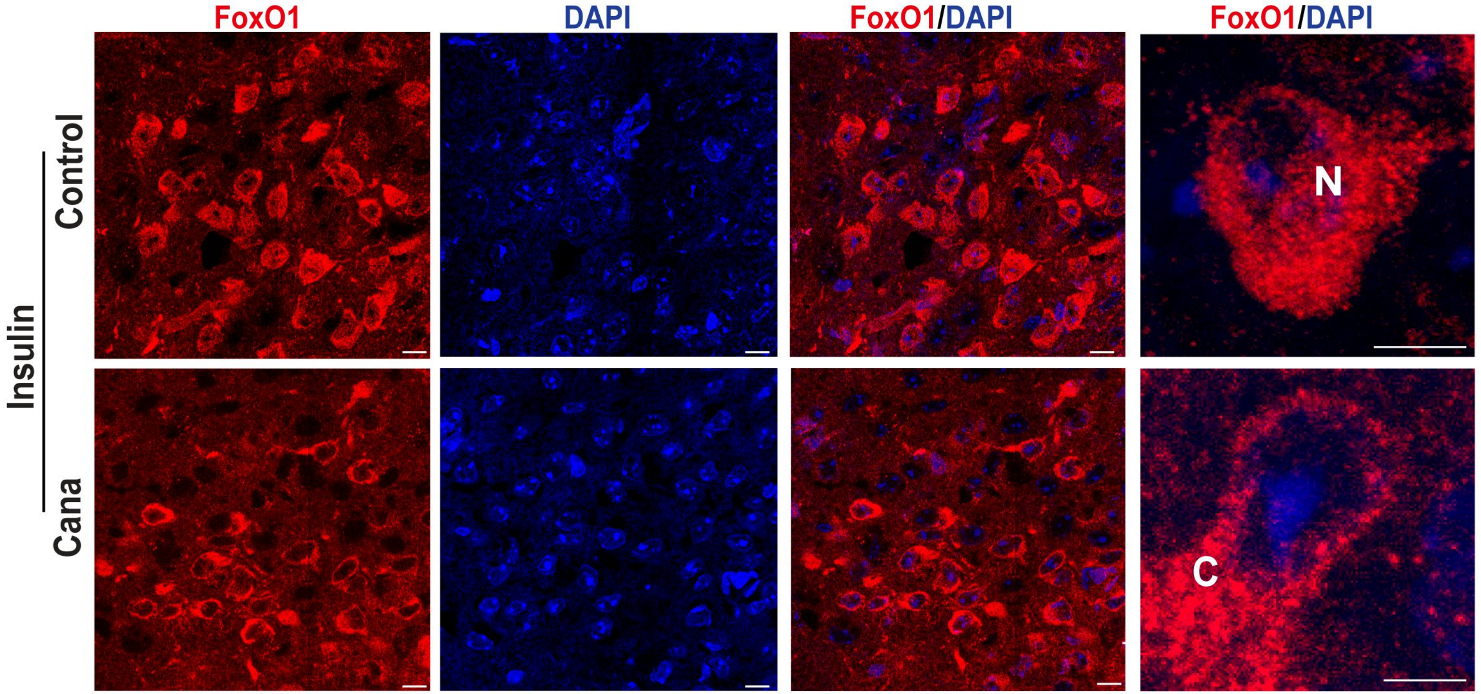

Supplementary Figure 2

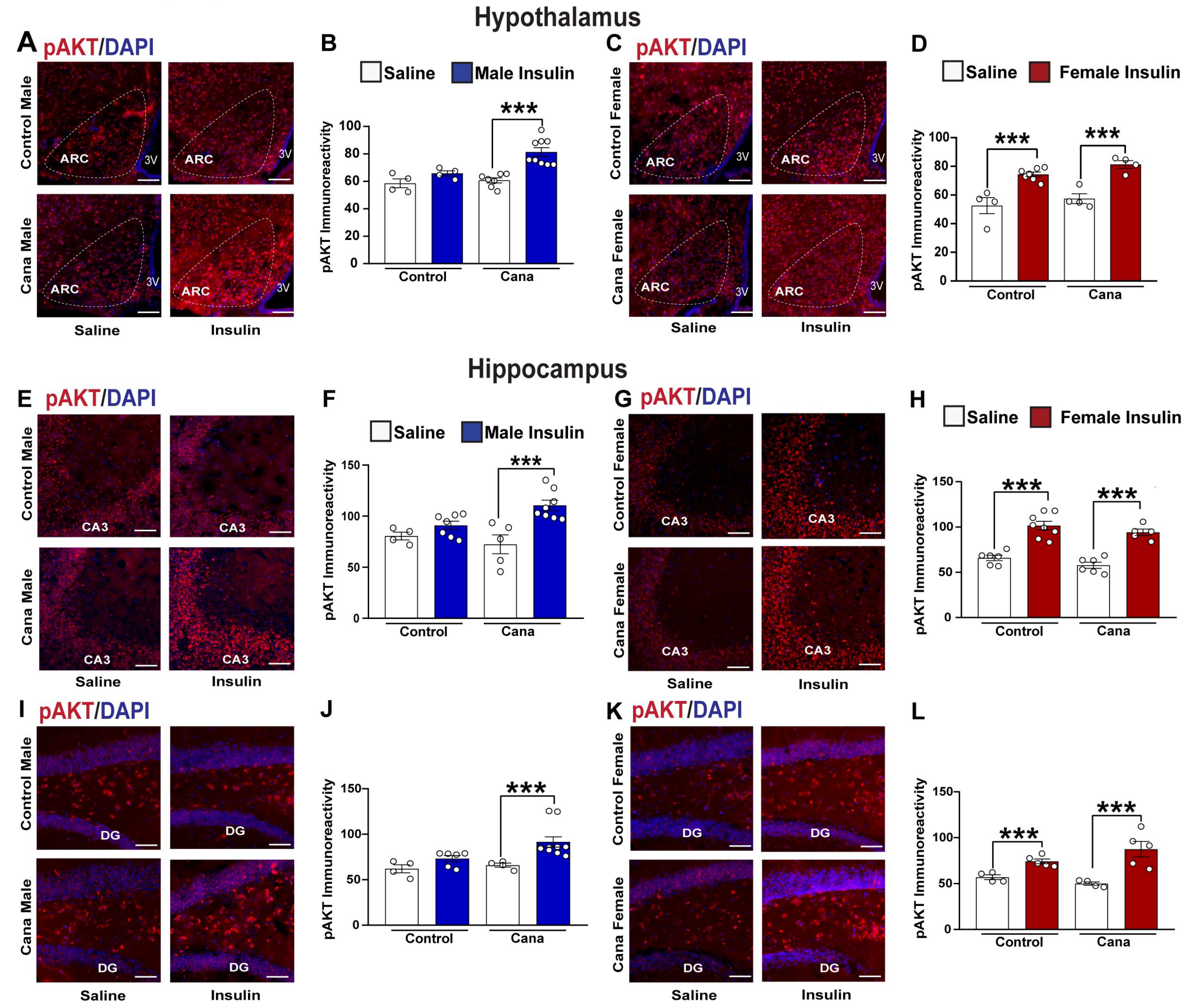

ANOVA	ARC	CA3	DG
Treatment	p < 0.05	p = 0.07	p = 0.35
Insulin	p < 0.001	p < 0.01	p < 0.001
Interaction	p < 0.05	p = 0.23	p < 0.05

ANOVA	ARC	CA3	DG
Treatment	p = 0.09	p = 0.55	p = 0.07
Insulin	p < 0.001	p < 0.001	p < 0.001
Interaction	p = 76	p = 0.07	p = 0.94

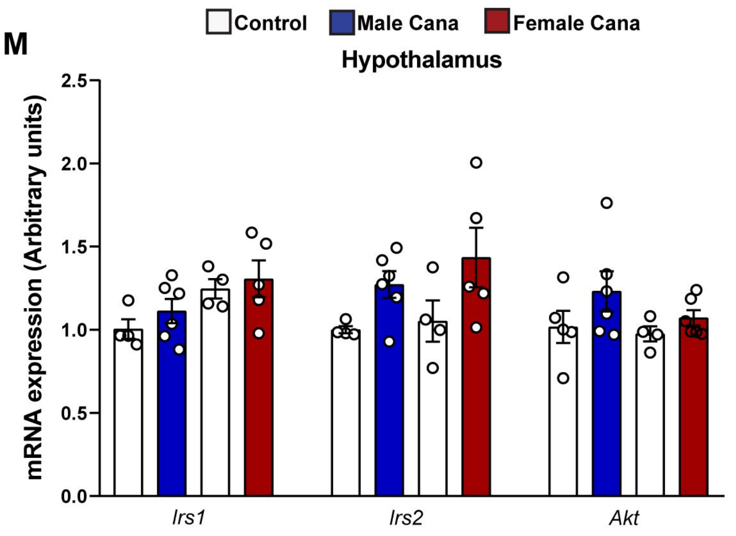

ANOVA	*Irs1*	*Irs2*	*Akt*
Treatment	p = 0.33	p < 0.05	p = 0.10
Sex	p < 0.05	p = 0.39	p = 0.28
Interaction	p = 0.78	p = 0.66	p = 0.52

Supplementary Figure 3

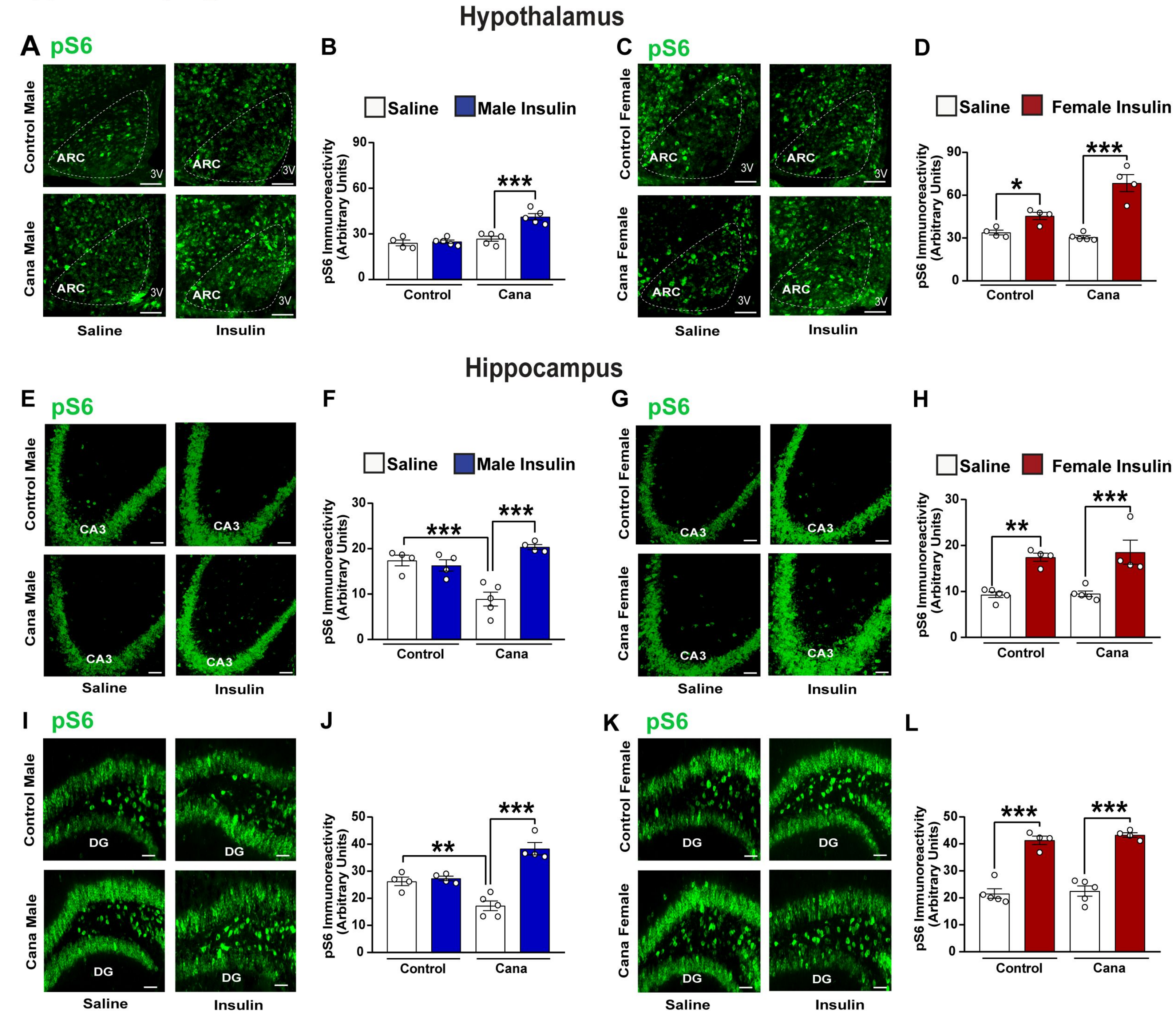

ANOVA	ARC	CA3	DG
Treatment	$p < 0.001$	$p = 0.10$	$p = 0.40$
Insulin	$p < 0.001$	$p < 0.01$	$p < 0.001$
Interaction	$p < 0.01$	$p < 0.001$	$p < 0.76$

ANOVA	ARC	CA3	DG
Treatment	$p < 0.01$	$p = 0.62$	$p = 0.40$
Insulin	$p < 0.001$	$p < 0.001$	$p < 0.001$
Interaction	$p < 0.01$	$p = 0.74$	$p = 0.76$

Supplementary Figure 4

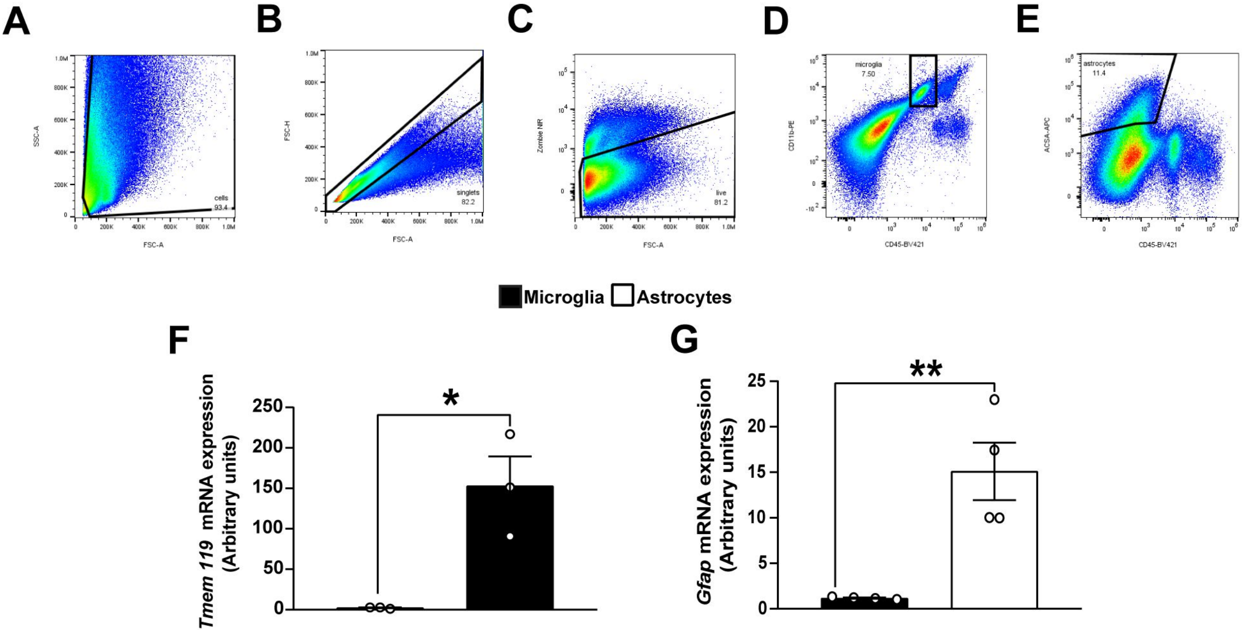

Supplementary Figure 5

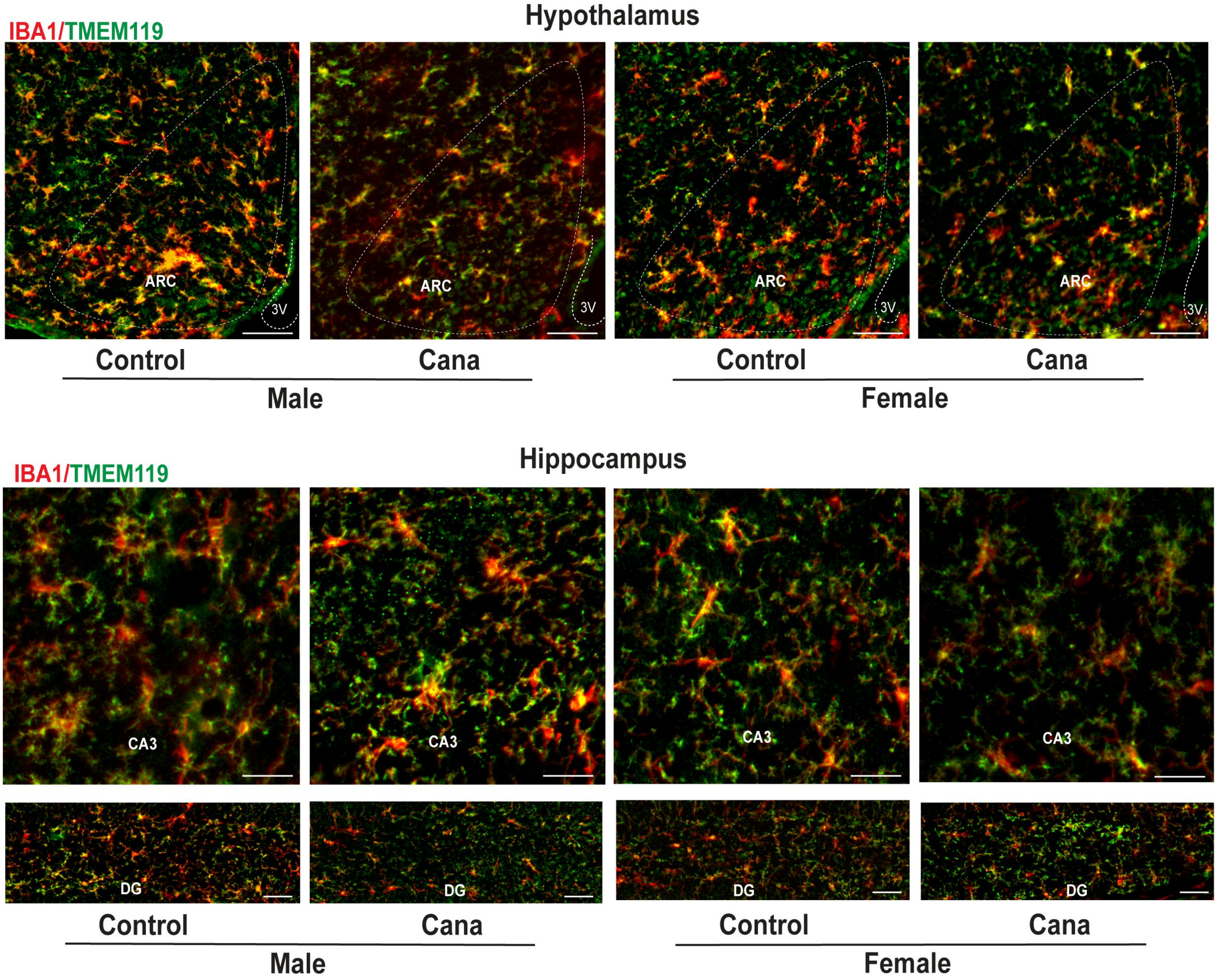

Supplementary Figure 6

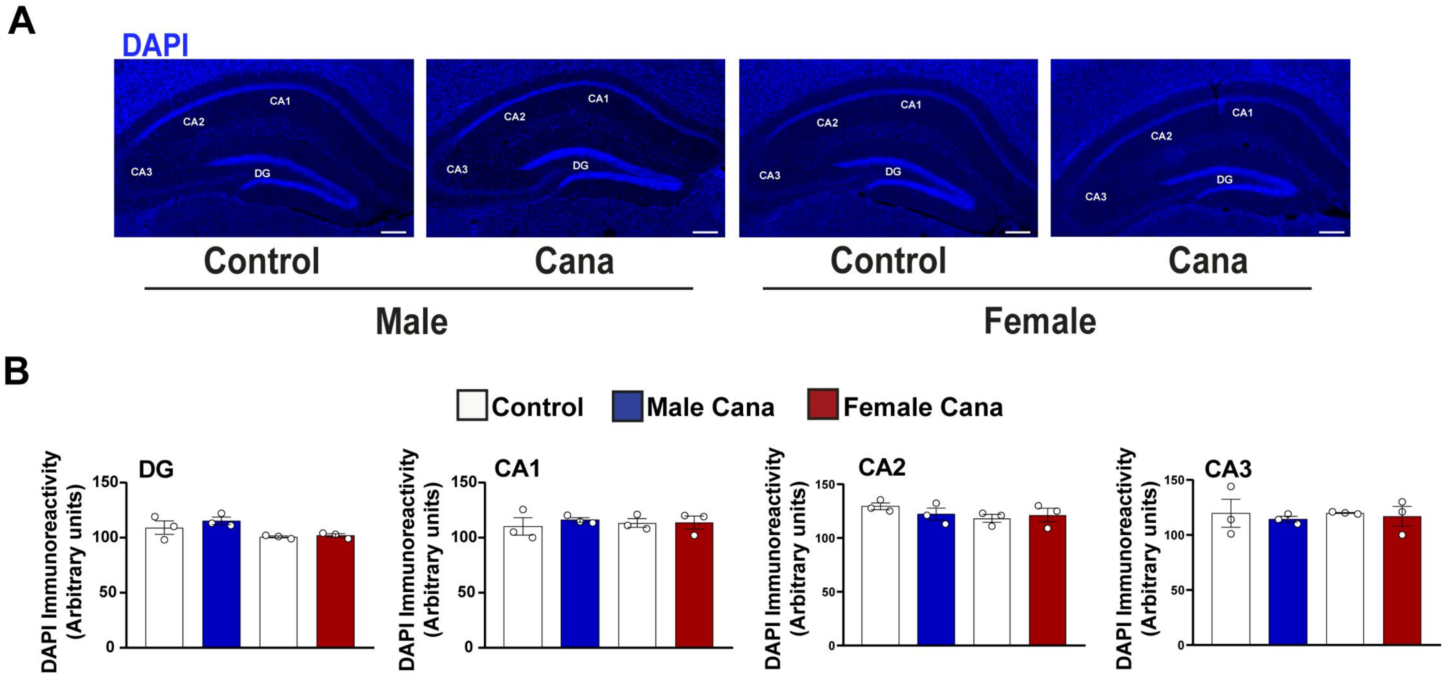

Supplementary Figure 7

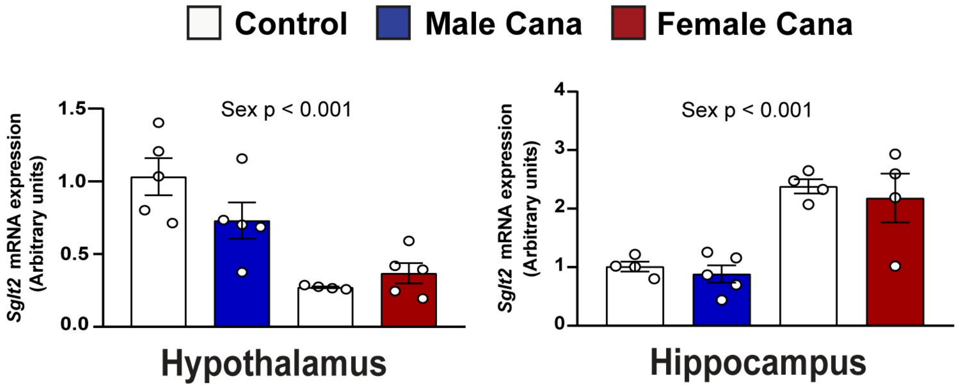

Supplementary Figure 8

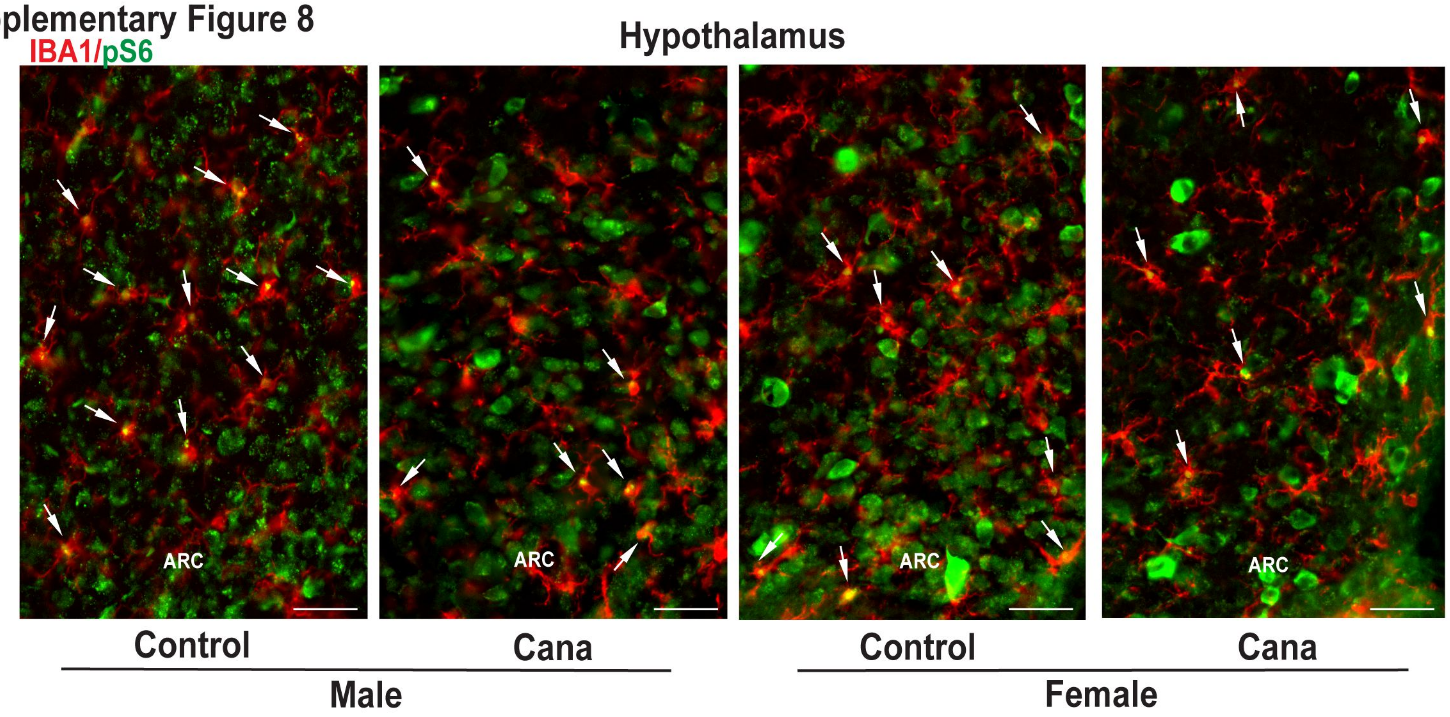

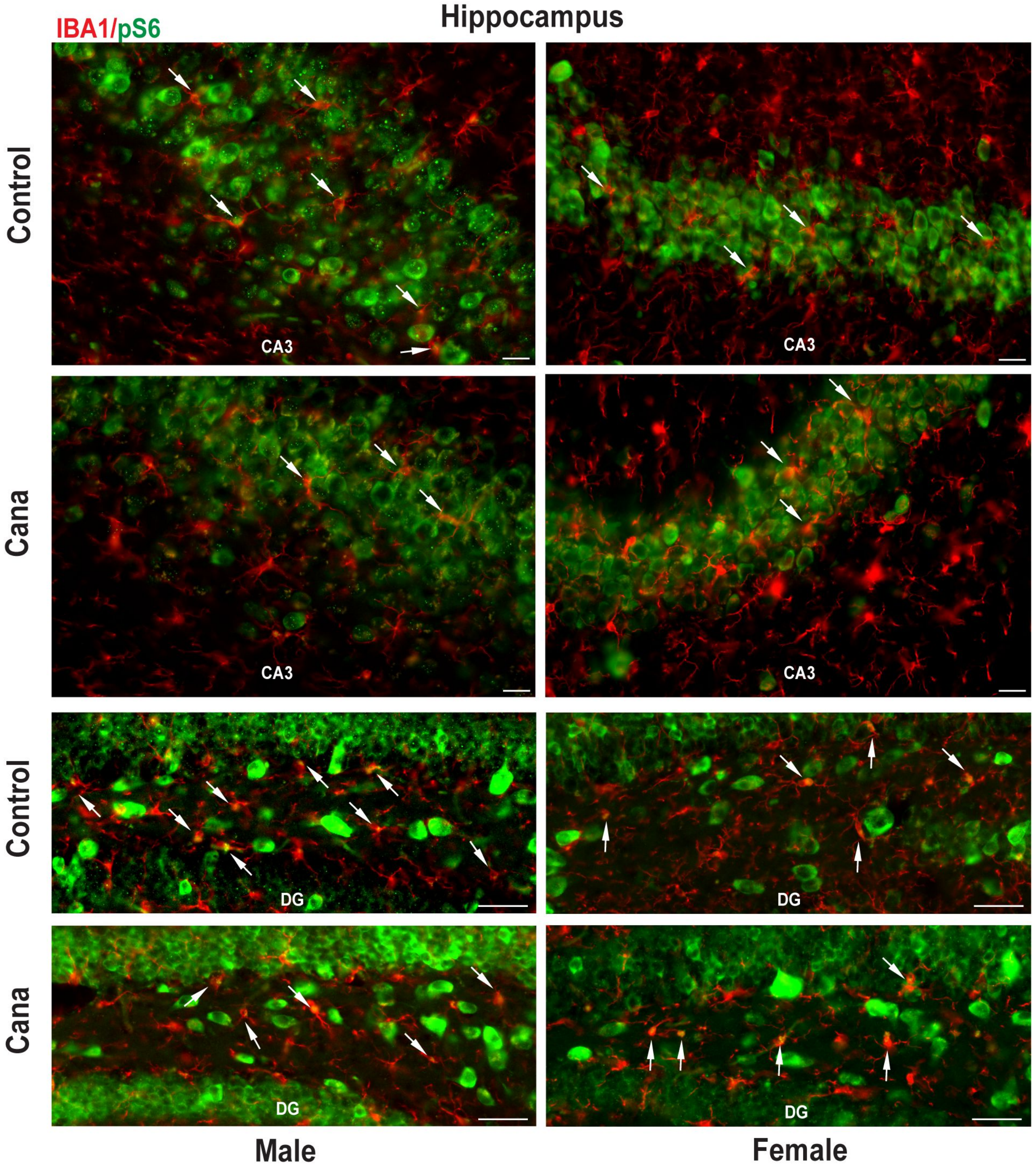

Supplement: Supplementary file 1 — Appendix S1 [file ACEL-21-e13653-s001.pdf]
